# A Case of Atraumatic Angular Vein Thrombosis

**DOI:** 10.7759/cureus.52465

**Published:** 2024-01-17

**Authors:** Sneha B Gajarla, Natalia Davila, Rodney Guiseppi, Mikelson MomPremier

**Affiliations:** 1 Department of Ophthalmology, University of Texas Medical Branch at Galveston, Galveston, USA

**Keywords:** lemierre's and lemierre's-like syndrome, virchow’s triad, thrombophlebitis, angular vein, thrombosis

## Abstract

Angular vein thrombosis is a rare vascular disorder marked by the occlusion of the angular vein by a blood clot. We report the case of a 70-year-old woman who presented with a visible bulge near her left nasojugal groove for one week and had no other associated symptoms or pertinent medical history. Subsequent maxillofacial CT scans with contrast revealed left angular vein thrombosis with dilation of the facial vein inferiorly. Despite its rarity, angular vein thrombosis poses a diagnostic challenge due to overlapping symptoms with other vascular and nonvascular pathologies. This case highlights the importance of early identification to prevent complications such as facial venous circulation obstruction and clot migration to the cavernous sinus.

## Introduction

Angular vein thrombosis is a rare disorder characterized by the formation of a blood clot in the angular vein [1]. The angular vein is located on the side of the root of the nose and is formed superiorly by the supraorbital and supratrochlear veins [2, 3]. It runs adjacent to the terminal branch of the facial artery, the angular artery [2, 3]. It extends inferiorly to form the facial vein, which drains into the cavernous sinus as well as the pterygoid plexus [2-4]. Therefore, the angular vein provides an important anastomosis between the facial vein and the cavernous sinus [2, 3]. Pathologies of the angular vein are rare and therefore can often be misdiagnosed as abnormalities of surrounding structures, such as the nasolacrimal system [5]. Identifying the disorder when it is presented is crucial to prevent complications of worsening obstruction of facial venous circulation and potential clot migration to the cavernous sinus [1].

Thrombosis is most common in deep veins, but its pathophysiology for superficial veins is still related to the underlying principle of Virchow’s triad [6]. Virchow’s principle describes the components that predispose to thrombosis, which include blood stasis, hypercoagulability, and vascular trauma [6-8]. Other etiologies have also been associated, such as mechanical injuries, cosmetic fillers, infections, predisposing thrombophilia, and other disorders [6-10]. Among the few reported cases of angular vein thrombosis, the common underlying clinical feature was a visible induration or mass. The symptoms reported include tearing and previous swelling. Common patient histories note previous surgeries near the area and nasolabial fillers. It is essential to differentiate between other disorders with similar clinical features, such as thrombophlebitis, pyogenic granulomas, and nasolacrimal obstruction [1, 11]. Further, Lemierre's syndrome has been reported to cause facial vein thrombosis and should be ruled out when diagnosing angular vein thrombosis [9, 12].

Considering angular vein thrombosis when evaluating patients with angular vein pathologies is essential. With less than 10 total cases reported in the literature [1, 5, 11, 13], there is a paucity of and a need for prior research to learn more about the condition of angular vein thrombosis. Here, we report a unique patient presentation of angular vein thrombosis.

## Case presentation

**Figure 1:**
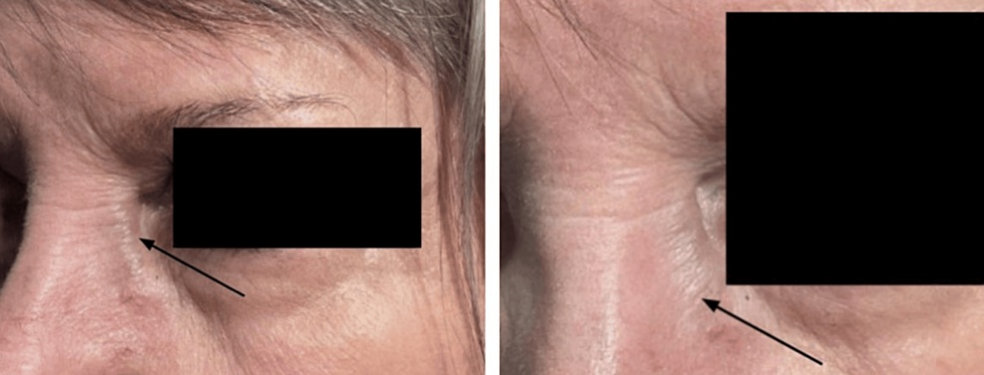
A distended left angular vein is seen on presentation.

A 70-year-old woman presented to the ophthalmology clinic following an emergency department admission for a bulge near her left lower lid (LLL) along the nasojugal fold area for one week with no associated pain, discharge, or recent trauma (Figure 1).

**Figure 2:**
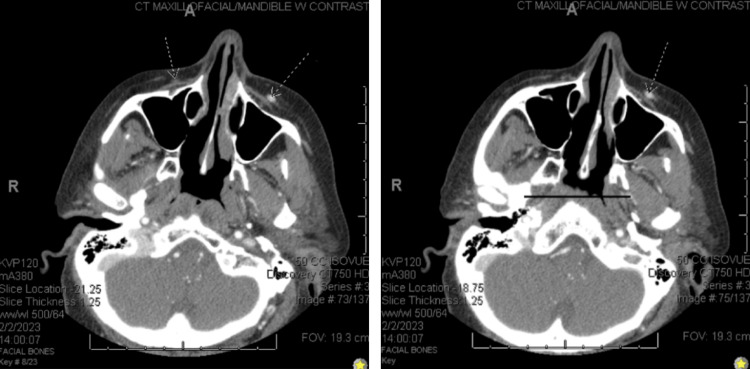
Two slides from the patient's CT with contrast of the maxillofacial/mandibles demonstrate a diffuse asymmetric prominence of the left facial vein when compared to the right facial vein. This indicates the presence of a varix on the left.

She had preserved extraocular motion, stable visual acuity, and no diplopia or conjunctival injection. The patient had no previous face, head, or neck surgeries. Significant ophthalmological history included dry eyes, laser-assisted in situ keratomileusis (LASIK), physiological anisocoria, and combined forms of age-related cataracts of both eyes. Significant examination findings included a prominent inferior angular vein on the left side and oval dilation of the left pupil without any ptosis or afferent pupillary defect with full extraocular motility. Vision and intraocular pressures were normal, and the patient had an unremarkable dilated fundus exam. A maxillofacial computed tomography (CT) scan with contrast revealed angular venous thrombosis of unknown acuity with an abrupt diminishment in the caliber of the superior palpebral vein and dilatation of the facial vein inferiorly (Figure 2).

An incidental finding of the retropharyngeal course of carotid arteries was also noted. The CT was negative for dilatation or thrombosis in the communicating danger zone or intraocular veins, including the lacrimal vein, superior ophthalmic vein, or cavernous sinus (Figure 2).

Ultimately, the diagnosis of left-sided angular venous thrombosis was made based on the CT findings. Laboratory studies of angiotensin-converting enzyme, serum lysozyme, syphilis immunoglobulin (Ig) G and IgM, anti-nuclear antibody screen, anti-neutrophil cytoplasmic antibody screen, sedimentation rate, c-reactive protein, rheumatoid factor, complete blood count with differential, complete metabolic panel, and lipid panel were all ordered and were unremarkable. The thrombophilia panel included prothrombin time, international normalized ratio, and activated partial thromboplastin time, which were all normal. The extensive thrombophilia panel could not be completed due to a lack of follow-up. Upon referral to vascular surgery, it was determined the patient did not need acute surgical intervention and should continue aspirin (81 mg) daily.

A follow-up of six months was made to check the patient’s eyelids, motility, and pupils, but the patient was lost to follow-up. The patient was advised to present to the emergency department if any changes in visual acuity, acute-onset diplopia, eye pain, or worsening of swelling were to occur.

## Discussion

Pathologies of the angular vein may lead to symptoms such as pain, swelling, skin discoloration, and vision changes. They can involve various pathological conditions, including vascular malformations [14] and tumors. Angular vein thrombosis, often underdiagnosed due to its rarity, presents unique challenges. This case and the few previously published describe angular vein thrombosis as a compressible swelling with associated tearing, but other symptoms are variable and generalizable. The compressibility of thromboses as angular vein thrombosis is likely seen proximal to the lesion due to the engorgement of the vein from the decreased outflow. Other important sources to consider include inflammatory and infectious etiologies such as thrombophlebitis [15] and Lemierre's syndrome [12], iatrogenic etiologies from previous surgeries (cosmetic filler injections), hypercoagulable and idiopathic causes, as well as nonvascular etiologies such as dacryocystitis, mucoceles, and malignancy. Angular vein thrombosis should be considered in patients presenting with a compressible tubular lesion in the nasojugal area. Distinguishing angular vein thrombosis from thrombophlebitis and Lemierre's syndrome is crucial. Thrombophlebitis exhibits localized signs [15], such as orbital swelling, maxillary tenderness, and erythema, whereas Lemierre's syndrome is an infectious thrombophlebitis that involves the internal jugular vein with a respiratory infection origin [12]. Angular vein thrombosis, centered around the eye, brings forth aesthetic concerns and potential vision changes [1].

Vascular malformations of the angular vein refer to abnormal formations of blood vessels in the region and include various etiologies, such as varix formation and cavernous venous malformation. Vascular tumors involving the angular vein include abnormal growths of blood vessels, including intravenous pyogenic granulomas and intravascular papillary endothelial hyperplasia [1]. Infections affecting the angular vein, such as thrombophlebitis, can occur due to the spread of microorganisms through the bloodstream or direct invasion from adjacent structures. Infections can cause localized inflammation, pain, and swelling in the affected area. This expanded perspective acknowledges the diverse factors that must be taken into consideration when diagnosing pathologies of the angular vein as well as ruling in or out an angular vein thrombosis.

With the plethora of conditions to consider that can occur in the angular veins, it is important to perform imaging to confirm or rule out pathologies in the broader differential. By utilizing imaging techniques such as ultrasound, CT, or MRI, healthcare professionals are provided with detailed anatomical information, allowing for a comprehensive assessment of the area. By visualizing the blood vessels and surrounding tissues, imaging can help differentiate between these possibilities and provide a more accurate diagnosis. This information is crucial for determining the most appropriate management and treatment options. Once the diagnosis of angular vein thrombosis can be confirmed with imaging, a hypercoagulable workup should be ordered for review. Table 1 describes all currently reported cases of angular vein thrombosis; it serves to demonstrate the various clinical findings and the imaging techniques used in making the diagnosis.

**Table 1:** List of all published cases of angular vein thromboses LASIK: laser-assisted in situ keratomileusis

Author	Patient details and history duration		History	Clinical findings	Treatment/ Diagnosis
Gajarla et al., 2024	70-year-old female; left	1 week	LASIK was done 20+ years ago. Painless bulging area and tearing right eye.	Left angular vein was distended inferiorly. MRI findings: diminishing caliber of the superior palpebral veins and dilations of facial vein inferiorly and anterior jugular vein. Retropharyngeal course of the carotid arteries	Thrombosis
Custer et al., 2023 [1]	44-year-old female; left	1 month	Left dacryocystorhinostomy was done 19 months ago. Swelling and tenderness.	Linear subcutaneous induration en route of the angular vein	Thrombosis
Custer et al., 2023 [1]	62-year-old female; left	9 months	Nasolabial filler one year before. Swelling and tenderness	Distended superiorly, induration and tenderness inferiorly. MRI findings: filler present near affected angular vein	Thrombosis
Imperial et al., 2006 [13]	56-year-old male; right	6 months	Compressible and non-tender mass superior to the medial canthal ligament	Thrombosed, isolated non-distensible angular vein varix	Varix thrombosis
Nasr & Huaman, 1998 [5]	35-year-old female; left	1 year	Intermittent swelling and tearing	Compressible mass, distention on Valsalva maneuver	Thrombosed varix
Khan et al., 2010 [11]	59-year-old female; left	6 months	Paranasal swelling, but with no associated epiphora, discharge, pain, or headache.	4 mm-5 mm, overlying the left medial canthal tendon. Emptied by firm pressure and readily refilled; it did not increase in size with the Valsalva maneuver	Varix
Khan et al., 2010 [11]	65-year-old female; right	4 years	Childhood pulmonary tuberculosis and paranasal lump increasing in size	Non-tender, purple, and mobile in the subcutaneous space; the lesion was not compressible, CT scan confirmed there was no associated intranasal or intraorbital anomaly	Thrombosis
Khan et al., 2010 [11]	61-year-old female; left	“Several” months	Painful initially; history of blepharitis and keratoconjunctivitis sicca secondary to glutaraldehyde exposure; no history of trauma to the area.	Non-tender, mobile in the subcutaneous space, and found to lie in the line of the angular vein; not compressible	Spontaneous thrombosis of varix

## Conclusions

While only seven reported cases of angular vein thrombosis existed prior to the current case, it is crucial to consider angular vein thrombosis as a potential diagnosis when evaluating patients with related symptoms. Moreover, the findings of this case bear broader implications for the fields of radiology and neurosurgery. Radiologists play a pivotal role in confirming angular vein thrombosis through imaging modalities, emphasizing the necessity for heightened awareness and inclusion of angular vein thrombosis in differential diagnoses when interpreting scans of the periorbital region. The collaboration between ophthalmologists and radiologists is vital for refining diagnostic protocols and ensuring accurate and timely diagnosis. As our knowledge of angular vein thrombosis expands, clinicians, particularly in neurosurgery, must be attuned to the potential involvement of the angular vein in patients presenting with periorbital symptoms. The intricate vascular anatomy of this region necessitates a multidisciplinary approach, involving neurosurgeons in the comprehensive assessment and management of angular vein thrombosis cases. This case report contributes to the limited literature on angular vein thrombosis, emphasizing the necessity of ongoing research to unravel the intricacies of angular vein pathologies and refine our approach to diagnosis and treatment. Ultimately, these advancements benefit medical practice by ensuring a holistic and collaborative perspective that spans ophthalmology, radiology, and neurosurgery, leading to more accurate diagnoses and targeted interventions for improved patient outcomes.

## References

[1] Custer PL, Ho TC, Boulos F (2023). Disorders of the angular vein. Ophthalmic Plast Reconstr Surg.

[2] René C (2006). Update on orbital anatomy. Eye (Lond).

[3] Azzam D, Cypen S, Tao J (2023). Anatomy, Head and Neck: Eye Ophthalmic Vein. https://www.ncbi.nlm.nih.gov/books/NBK557603/.

[4] Zhang J, Stringer MD (2010). Ophthalmic and facial veins are not valveless. Clin Exp Ophthalmol.

[5] Nasr AM, Huaman AM (1998). Anterior orbital varix presenting as a lacrimal sac mucocele. Ophthalmic Plast Reconstr Surg.

[6] Cosmi B (2015). Management of superficial vein thrombosis. J Thromb Haemost.

[7] Cushman M (2007). Epidemiology and risk factors for venous thrombosis. Semin Hematol.

[8] Ashorobi D, Ameer MA, Fernandez R (2023). Thrombosis. https://www.ncbi.nlm.nih.gov/books/NBK538430/.

[9] de Almeida MJ, Guillaumon AT, Miquelin D (2019). Guidelines for superficial venous thrombosis. J Vasc Bras.

[10] Pimentel de Miranda A, Nassiri N, Goldberg RA (2016). Engorgement of the angular and temporal veins following periorbital hyaluronic acid gel injection. Ophthalmic Plast Reconstr Surg.

[11] Khan SR, Burton BJ, Beaconsfield M, Rose GE (2004). The varix of angular vein. Eye (Lond).

[12] Cloutet A, Botta RK, Kulkarni SR, Ponna PK (2022). A complex case of Lemierre’s syndrome with facial vein involvement. Cureus.

[13] Imperial MT, Cheah E, Fong KS (2006). An unusual clinical presentation of angular vein varix. Orbit.

[14] Yang Y, Ye X, Fu B, Li Z, Feng Y, Zhao Y, Liu H (2021). Intravenous pyogenic granuloma in the internal jugular vein: a case report and literature review. Medicine (Baltimore).

[15] Ferris KP (1964). Angular vein thrombophlebitis-report of a case. J Laryngol Otol.

